# Citrulline inhibits LPS‐induced pyroptosis of RAW264.7 macrophages through NF‐κB signaling pathway

**DOI:** 10.1002/iid3.832

**Published:** 2023-04-19

**Authors:** Li Yin, Xiaomin Wei, Yanjun Zhang, Chengshu Lu, Huakun Wang

**Affiliations:** ^1^ Department of Biopharmaceutics Yulin Normal University Yulin China; ^2^ Bioengineering & Technology Center for Native Medicinal Resources Development Yulin Normal University Yulin China; ^3^ College of Pharmacy and Traditional Chinese Medicine Jiangsu College of Nursing Huai'an City Jiangsu Province China

**Keywords:** citrulline, macrophage, NF‐κB, p65, pyroptosis

## Abstract

**Background:**

The aim of this study was to investigate the effect of citrulline on the pyroptosis of mouse macrophage RAW264.7 and the mechanism. We investigated the effect of citrulline on pyroptosis of RAW264.7 cell induced by lipopolysaccharide (LPS), and the modulation of nuclear factor‐kappaB (NF‐κB) signaling.

**Methods:**

Pyroptosis was evaluated using flow cytometry and caspase‐1/sytox double staining. Cell counting kit‐8 assay was performed to evaluate cell viability.

**Results:**

Citrulline promoted cell viability and inhibited the pyroptosis of RAW264.7 cell stimulated by LPS. Furthermore, citrulline inactivated NF‐κb/p65 signaling pathway by suppressing p65 nuclear translocation induced by LPS. An NF‐κb signaling pathway activator, betulinic acid, reversed the inhibition of pyroptosis induced by citrulline.

**Conclusion:**

Citrulline inhibited LPS‐induced pyrophosis, which may be closely related to the inactivation of NF‐κB/p65 signaling pathway.

## Graphical abstract

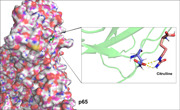

## 1 INTRODUCTION

Inflammation is a defensive response to various inflammatory factors and injuries.^1^, ^2^ Severe or persistent inflammation is the basis of some diseases.^3^, ^4^ Pyroptosis is a newfound programmed cell death, which is the result of activation of intracellular inflammasome.^5^ The occurrence of pyroptosis depends on activation of caspase‐1, and is closely related to diverse disorders.^6^, ^7^, ^8^ Therefore, regulating pyroptosis may be a method for targeted therapy of inflammation.

Citrulline is an amino acid originally found in watermelon. Citrulline is synthesized by ornithine carbamoyl transferase (OCT) and participates in the urea cycle under the catalysis of arginine succinyl synthetase (ASS). In nitric oxide (NO) producing cells, citrulline is involved in arginine synthesis through ASS, and arginine is further synthesized into NO, forming the citrulline‐NO‐cycle. NO is an important inflammatory factor that will increase the cardiovascular burden when excess. Citrulline regulates the synthesis of NO in this cycle through negative feedback, which can reduce the organ damage induced by oxidative stress.

Macrophages are the basic innate immune cells of inflammation. At the onset of inflammation, monocytes differentiate into macrophages, which promote inflammation to fight off pathogen invasion. On the other hand, macrophages prevent excessive inflammation.^9^ Pro‐inflammatory signals induce macrophage polarized into an M1 phenotype that promotes inflammation progression, whereas anti‐inflammatory signals induce an M2 phenotype that inhibits inflammation.^10^ The nuclear factor‐kappaB (NF‐κB) signaling pathway is considered a pro‐inflammatory pathway and has also been found to have anti‐inflammatory effects in recent studies.^11^ This pathway is commonly activated in microphages during inflammation.^12^ Hence, it is essential to explore the effects of citrulline on microphages pyroptosis through the NF‐κB pathway.

In this study, we investigated the effects of citrulline on the pyroptosis of macrophages induced by lipopolysaccharide (LPS) and the mechanism. We hope these findings will provide new ideas for the treatment of inflammation.

## 2 MATERIALS AND METHODS

### 2.1 Cell culture and treatment

RAW264.7 cells were purchased from the Cell Bank of the Institutes for Biological Sciences, and maintained in Dulbecco's modified Eagle medium (DMEM; HyClone) supplemented with 10% fetal bovine serum (FBS; Gibco) at 37°C with 5% CO_2_.

### 2.2 Flow cytometry assay

Cell pyroptosis was measured by flow cytometry assay with a FAM‐FLICA caspase‐1 kit (MBS258406; AmyJet Scientific) and Sytox Green reagent (C1070S; Beyotime). RAW264.7 cells were seeded into six well‐plates, and cultured for 24 h. Then the cells were treated with LPS (0, 1, 10, 100 ng/mL; Beyotime), citrulline (80 μM), and betulinic acid (BA; 10 µM, S0949; Selleck) for 4 h. Afterward, pretreated RAW264.7 cells were incubated with FAM‐YVAD‐FMK solution at 37°C in the dark for 1 h. The RAW264.7 cells were then stained with 1 mM Sytox Green for 10 min. Subsequently, the stained RAW264.7 cells were detected with flow cytometry (BD).

### 2.3 Cell viability assay

RAW264.7 cells were resuspended at 1 × 10^5^ cells/mL and then seeded in 96‐well plates. Cells were cotreated with citrulline (10, 20, 40, 80, and 160 μM) and LPS (100 ng/mL) at the same time for 24 h. Subsequently, 10 μL of cell counting kit‐8 (CCK‐8) reagents (AmyJet Technology) were added to each well and cultured in the incubator at 37°C for 4 h. Moreover, OD value under 450 nm wavelength was detected using a microplate reader (Molecular Devices).

### 2.4 DAPI/PI double satining

4',6‐diamidino‐2‐phenylindole/propidium iodide (DAPI/PI) double satining was performed to evaluate cell death.^13^ RAW264.7 cells were suspended with 1 mL cell staining buffer, followed by staining with 5 μL DAPI solution (C1005; Beyotime) and 5 μL PI staining solution (ST511; Beyotime), at 4°C for 30 min. Finally, the stained cells were observed under a fluorescence microscope (TE2000; Nikon).

### 2.5 Western blot

Primary antibodies used in this study were anti‐NLRP3 (ab263899, 1:800; Abcam), anti‐ASC (ab175449, 1:500; Abcam), anti‐caspase‐1 (ab138483, 1 µg/mL; Abcam), anti‐N‐GSDMD (ab215203, 1:800; Abcam), anti‐inhibitor a of NF‐κB (IκBα) (#4814S, 1:800; Cell Signaling Technology), anti‐p‐IκBα (#2859, 1:800; Cell Signaling Technology), anti‐p65 (ab32536, 1:800; Abcam), LaminB1 (ab16048, 0.1 µg/mL; Abcam), and anti‐GAPDH (ab8245, 1:1000; Abcam). The nuclear and cytoplasmic proteins from RAW264.7 cells were isolated with a nuclear extraction kit (P0027; Beyotime). The total protein concentration in each cell lysate was determined with a BCA Protein Assay Kit (Beyotime). Subsequently, the proteins were separated by 10% sodium dodecyl sulfate‐polyacrylamide gel electrophoresis and electrotransferred to polyvinylidene fluoride (PVDF) membranes. The membranes were blocked with 5% skim milk for 60 min and incubated with primary antibodies at 4°C overnight. Next day, the membranes were incubated with secondary antibodies (ab6721, 1: 2000; Abcam) at room temperature for 1.5 h. Finally, the protein expression was determined by an ECL kit and Scion Image v. 4.0.2 software (Scion Corporation).

### 2.6 Immunofluorescent staining

RAW264.7 cells were seeded in 24‐well plates at 1.8 × 10^5^ cells/well, and fixed with 4% paraformaldehyde for 15 min. Then 1 mL 0.05% Triton X‐100 was added and incubated for 5 min at 4°C. After blocking with 5% bovine serum albumin (Sigma) for 2 h, the cells were hybridized with anti‐p65 (ab32536, 1:100; Abcam) at 4°C overnight. Afterward, the cells were stained with DAPI for 10 min and imaged by a laser confocal microscope (GE Healthcare).

### 2.7 Bioinformatic analysis

Molecular docking was conducted to showed that the combination between citrulline and p65. Protein crystal structure of p65 was downloaded from RCSB PDB database (https://www1.rcsb.org/) and the pdb file was obtained. AutoDock Tools was used to process protein crystals and compound structures, including separating protein structures and original ligands, removing water molecules, adding charges to structures. Finally, the files were converted into PDBQT format and molecular docking was conducted. The docking results are visualized by PyMOL software.

### 2.8 Statistical analysis

SPSS v.19.0 (IBM, SPSS) was used for statistical analysis. Data were presented as means ± SD. The difference was compared using one‐way analysis of variance (ANOVA) followed by Tukey's post hoc test. *p* < .05 was considered statistically significant. Each assay was performed in triplicate.

## 3 RESULTS

### 3.1 LPS induces pyroptosis of RAW264.7 macrophages

**Figure 1:**
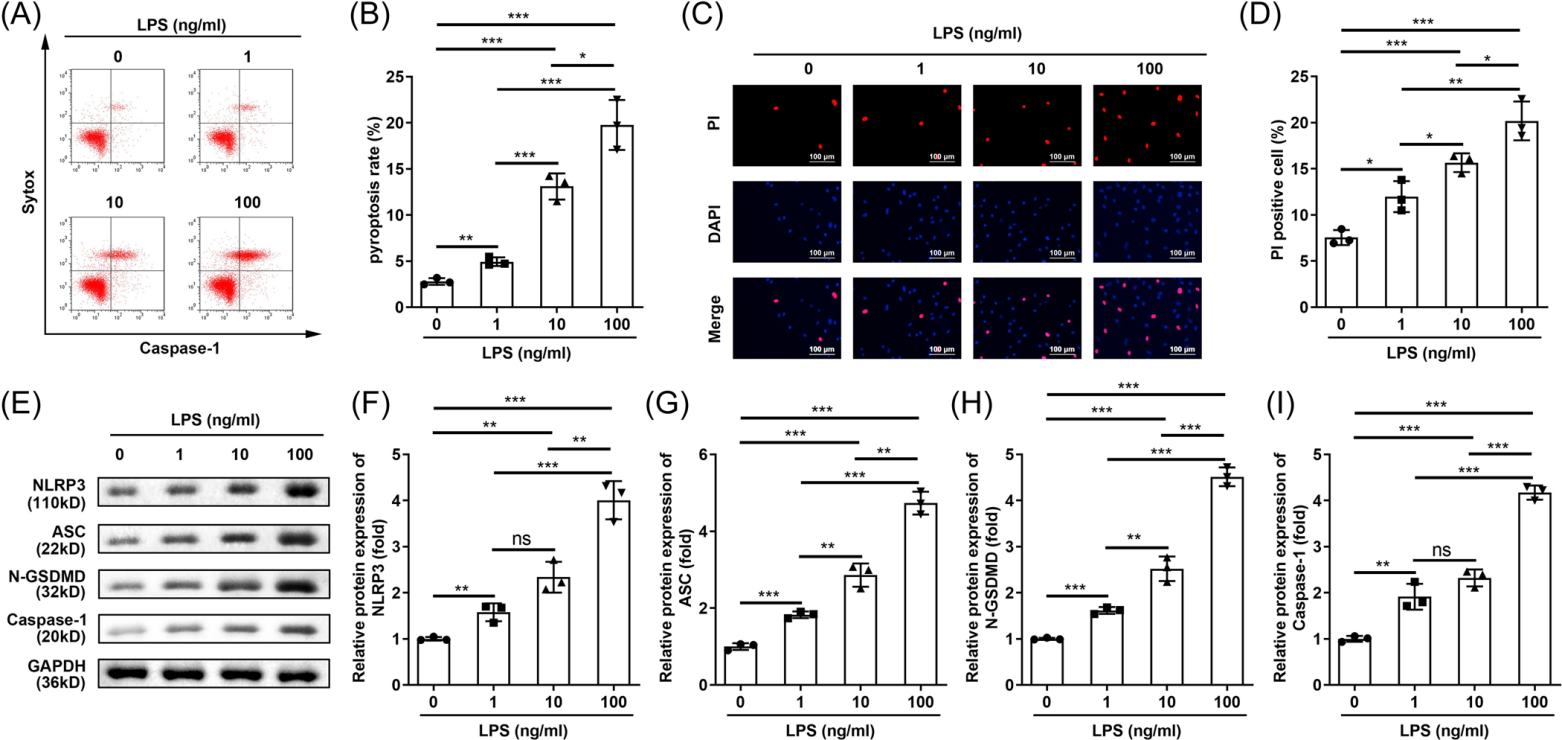
LPS induces pyroptosis of RAW264.7 macrophages. (A, B) Caspase‐1/sytox green staining and flow cytometry was used to detect the pyroptosis of LPS‐treated RAW264.7 cells. (C, D) DAPI/PI double staining assay was performed to detect cell death (magnification: ×100). (E) Western blot analysis was used to evaluate the expression of NLRP3, ASC, N‐GSDMD, and caspase‐1 in LPS‐treated RAW264.7 cells. *N* = 3. Data were analyzed using one‐way ANOVA followed by Tukey's test Protein levels of (F) NLRP3, (G) ASC, (H) N‐GSDMD, and (I) caspase‐1 were quantified. *p < .05, **p < .01, ***p < .001. ANOVA, analysis of variance; LPS, lipopolysaccharide.

First, cell pyroptosis of RAW264.7 cells treated with LPS was measured by flow cytometry assay, the results demonstrated that LPS prominently increased the pyroptosis in a dose‐dependent form (Figure 1A,B). DAPI/PI double satining also illustrated that PI‐stained cells were increased by LPS (Figure 1C,D). Meanwhile, the protein levels of NLRP3, ASC, N‐GSDMD, and caspase‐1 in RAW264.7 macrophages stimulated LPS were remarkably increased in a dose‐dependent form (Figure 1E–I). Thus, 100 ng/mL LPS was selected for the subsequent induction of pyroptosis.

### 3.2 Citrulline inhibits pyroptosis of RAW264.7 macrophages stimulated by LPS

**Figure 2:**
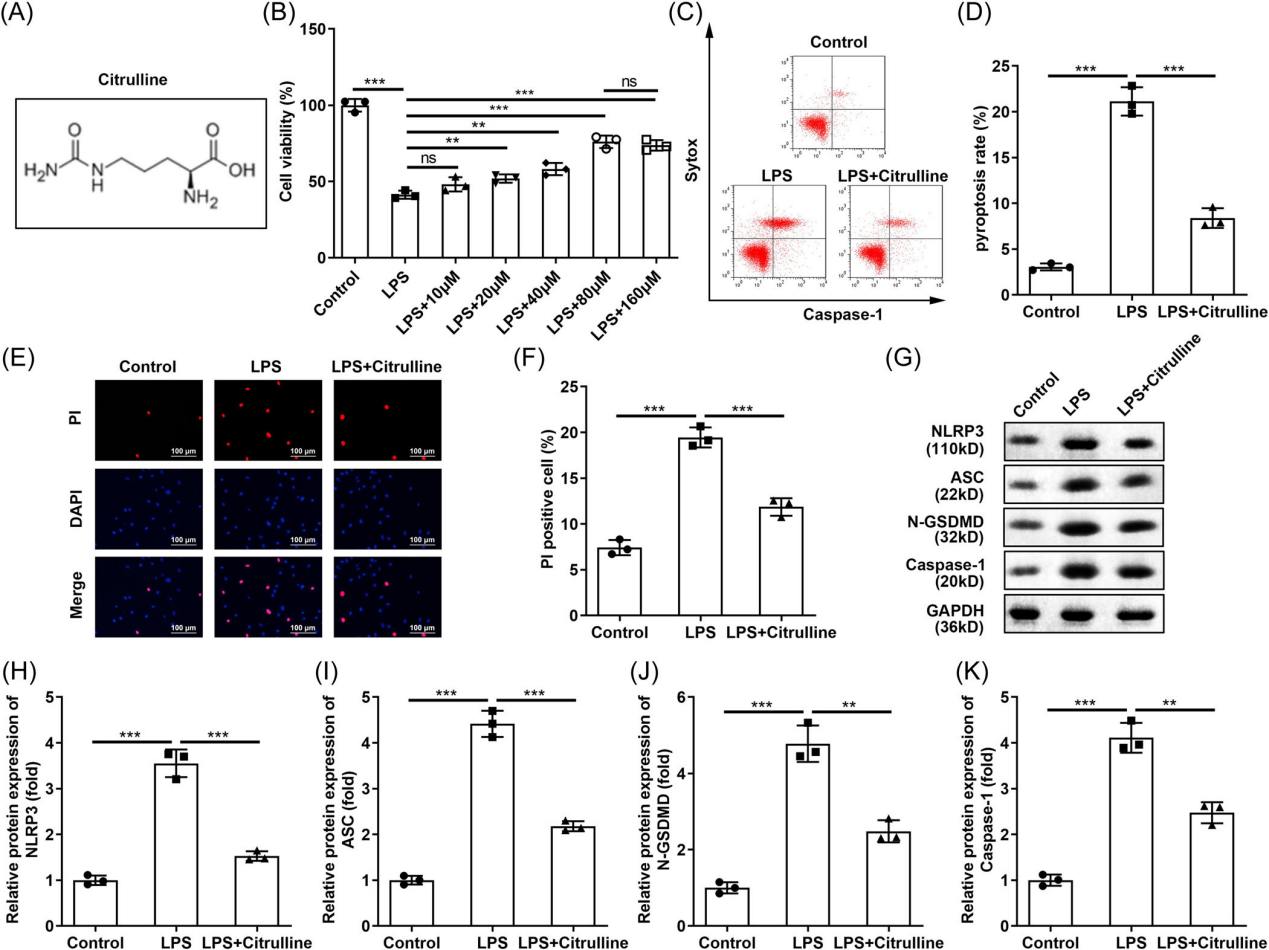
Citrulline inhibits pyroptosis of RAW264.7 macrophages stimulated by LPS. Molecular structure of citrulline. (B) Cell viability of RAW264.7 cells treated with LPS and citrulline was measured by CCK‐8 assay. (C, D) Cell pyroptosis of RAW264.7 cells treated with LPS and citrulline was evaluated by flow cytometry. (E, F) Cell death of RAW264.7 cells treated with LPS and citrulline was evaluated by DAPI/PI double staining assay (magnification: ×100). (G–K) NLRP3, ASC, N‐GSDMD, and caspase‐1 protein levels in RAW264.7 cells treated with LPS and citrulline were measured by western blot. *N* = 3. Data were analyzed using one‐way ANOVA followed by Tukey's test. **p < .01, ***p < .001. ANOVA, analysis of variance; LPS, lipopolysaccharide, ns, no statistical.

As indicated by Figure 2A, citrulline is an alpha‐amino acid (C_6_H_13_N_3_O_3_). RAW264.7 macrophages were treated with the indicated concentrations of citrulline (10–160 µM) under the stimulation of LPS. Cell viability of LPS‐treated RAW264.7 cells was dramatically increased by citrulline according to CCK‐8 assay, and the 80 µM citrulline was selected for the following experiments (Figure 2B). Moreover, cell pyroptosis of RAW264.7 cells treated with LPS was dramatically inhibited according to the flow cytometry and DAPI/PI double staining assays (Figure 2C–F). Besides, the protein levels of NLRP3, ASC, N‐GSDMD, and caspase‐1 in RAW264.7 macrophages stimulated LPS were remarkably decreased by citrulline treatment (Figure 2G–K).

### 3.3 Citrulline suppresses NF‐κB/p65 nuclear translocation in RAW264.7 macrophages induced by LPS

**Figure 3:**
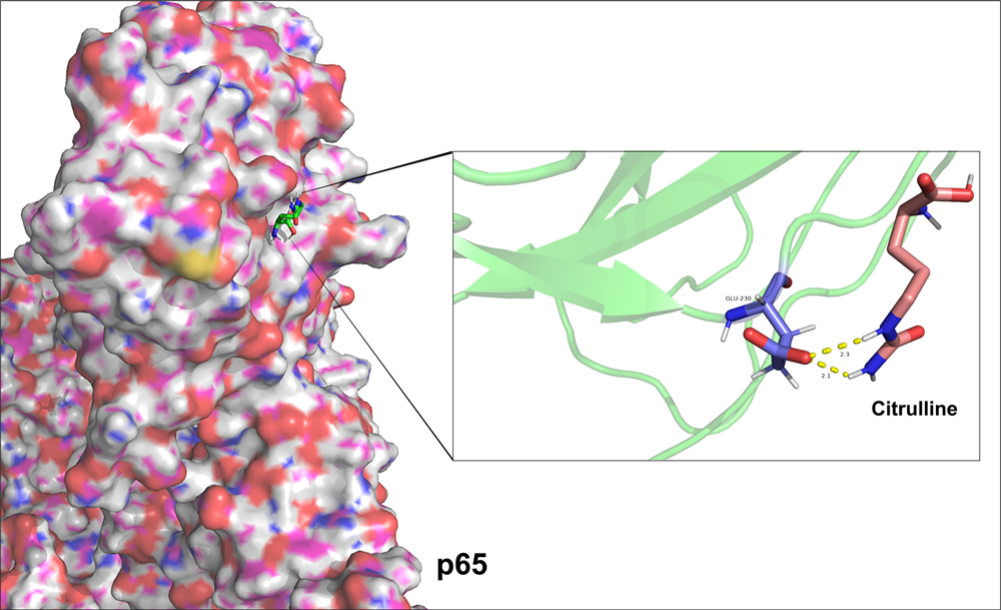
Molecular docking between citrulline and p65.

**Figure 4:**
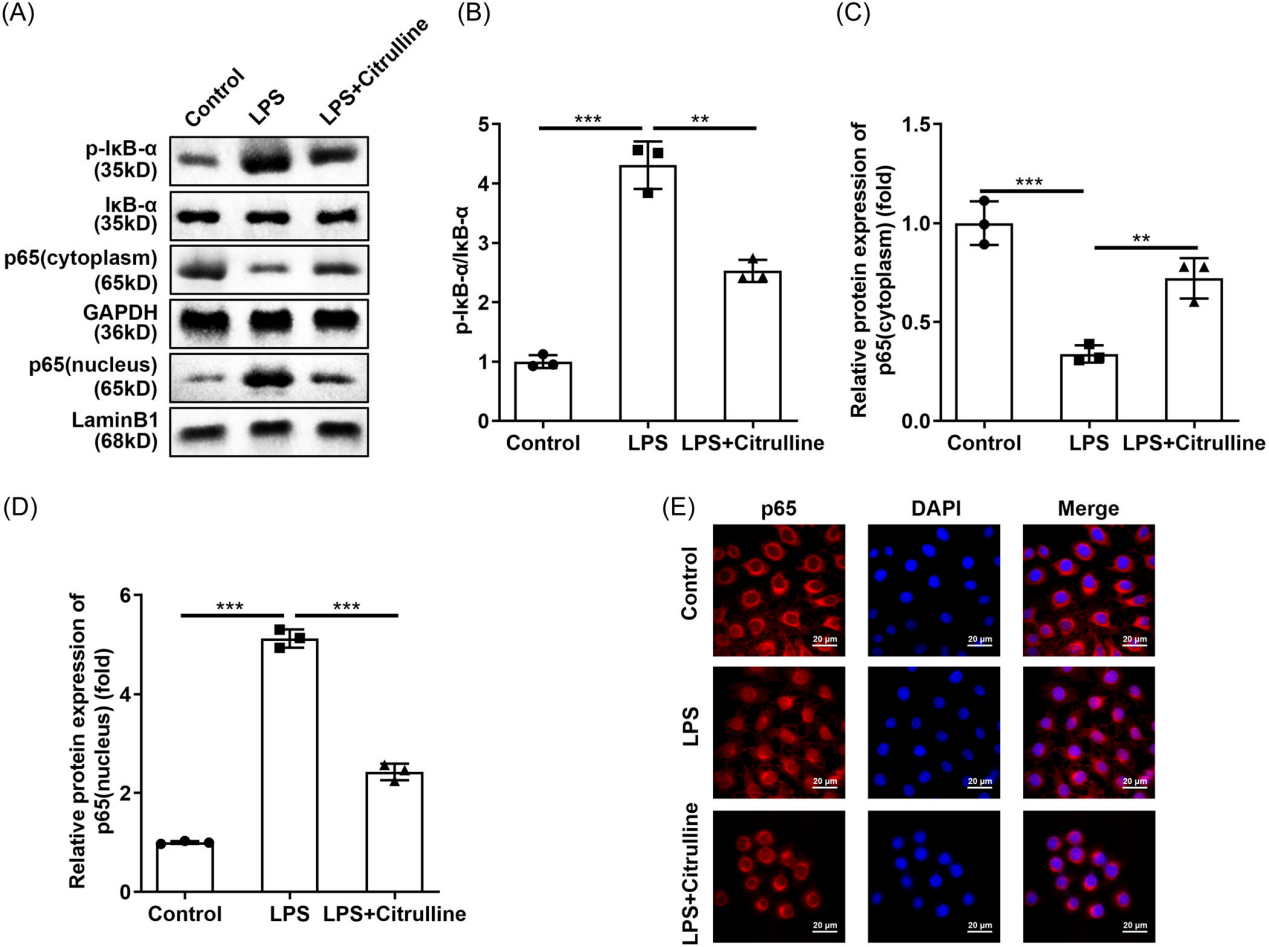
Citrulline suppresses NF‐κB/p65 nuclear translocation in RAW264.7 macrophages induced by LPS. (A–D) The levels of IκB‐α and p65 and their phosphorylated form proteins were quantified by western blot in RAW264.7 cells treated with LPS and citrulline. (E) The protein level of p65 in RAW264.7 cells treated with LPS and citrulline was assessed by immunofluorescence staining (magnification: ×400). *N* = 3. Data were analyzed using one‐way ANOVA followed by Tukey's test. **p < .01, ***p < .001. ANOVA, analysis of variance; LPS, lipopolysaccharide; NF‐κB, nuclear factor‐kappaB.

The molecular docking showed that the citrulline have good combination with the p65 target (Figure 3), indicating that citrulline may regulate p65‐related pathways. Western blot results revealed that LPS enhanced NF‐κB/p65 activation, while citrulline decreased the activation (Figure 4A,B). To further investigate whether citrulline modulates NF‐κB/p65 signaling pathway, the nuclear and cytoplasmic expression of p65 was measured. The results demonstrated that LPS promotes the nucleus translocation of p65, whereas citrulline prominently reversed this translocation process (Figure 4C,D). Meanwhile, p65 localization in RAW264.7 cells by immunofluorescence assay was performed, and the images also showed that p65 was enriched in the cytoplasm of normal RAW264.7 cells, but after LPS treatment, p65 was translocated to the nucleus, while citrulline significantly slowed down this process (Figure 4E).

### 3.4 Citrulline suppresses pyroptosis of RAW264.7 macrophages stimulated by LPS through inactivating NF‐κB/p65 pathway

**Figure 5:**
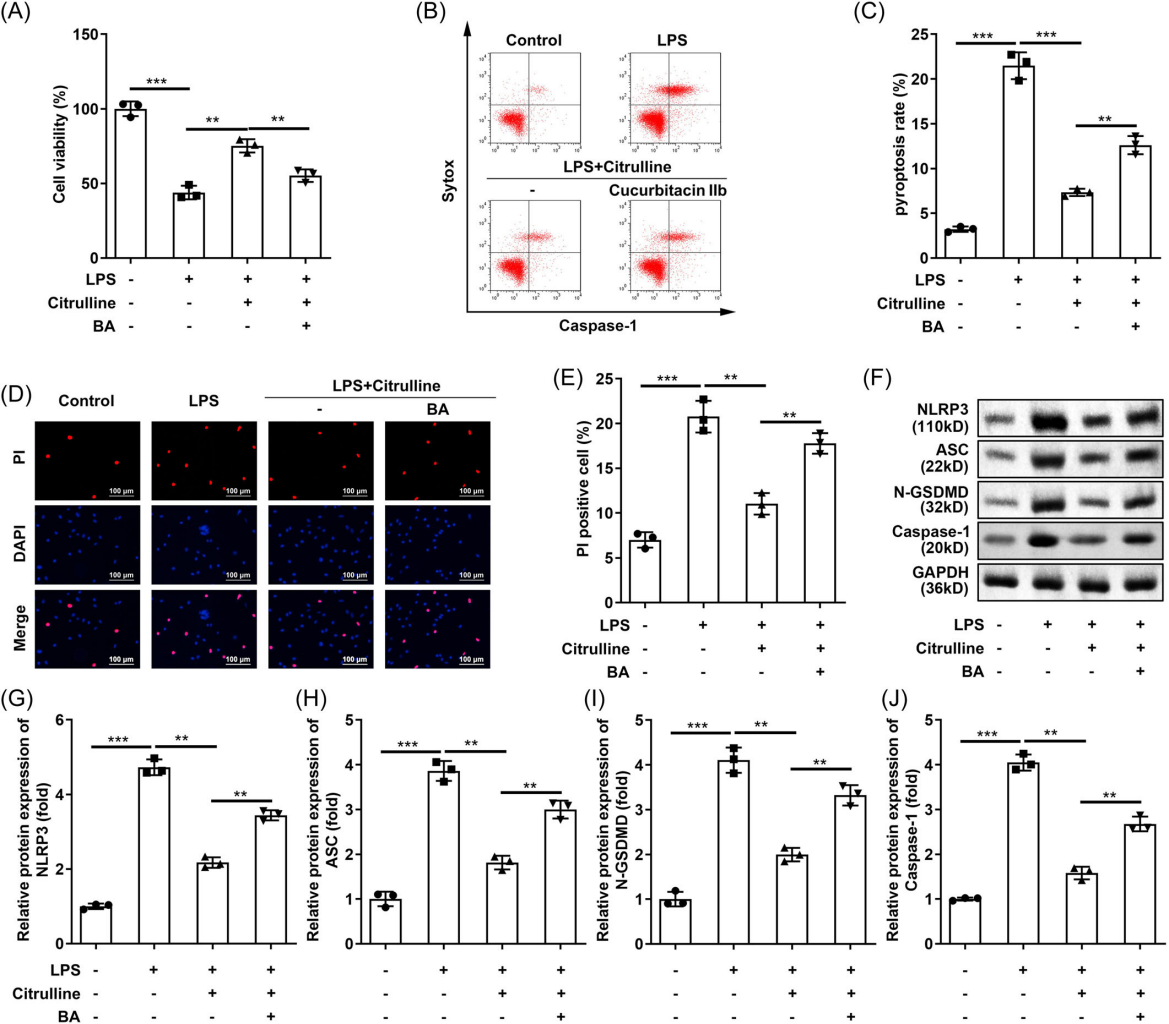
Citrulline suppresses pyroptosis of RAW264.7 macrophages stimulated by LPS by inactivating NF‐κB/p65 pathway. (A) Cell viability of RAW264.7 cells treated with LPS, citrulline, and BA was measured by CCK‐8 assay. (B, C) Cell pyroptosis of RAW264.7 cells treated with LPS, citrulline, and BA was evaluated by flow cytometry. (D, E) Cell death of RAW264.7 cells treated with LPS, citrulline, and BA was evaluated by DAPI/PI double staining assay (magnification: ×100). (F–J) NLRP3, ASC, N‐GSDMD, and caspase‐1 protein levels in RAW264.7 cells treated with LPS, citrulline, and BA were measured by western blot. *N* = 3. Data were analyzed using one‐way ANOVA followed by Tukey's test. **p < .01, ***p < .001. ANOVA, analysis of variance; BA, betulinic acid; LPS, lipopolysaccharide; NF‐κB, nuclear factor‐kappaB.

BA, which can activate NF‐κB signaling, was used to verify whether citrulline could suppress pyroptosis through NF‐κB/p65 pathway. As speculated, BA dramatically inhibited cell viability of citrulline treated RAW264.7 cells (Figure 5A). Simultaneously, pyroptosis cells were remarkably increased after BA treatment compared with the citrulline group (Figure 5B–E). Afterward, the protein levels of NLRP3, ASC, N‐GSDMD, and caspase‐1 in RAW264.7 macrophages decreased by citrulline were all prominently elevated by BA (Figure 5F–J).

## 4 DISCUSSION

In the current study, the results indicated that citrulline suppressed LPS‐induced RAW264.7 pyroptosis via inactivating the NF‐κB/p65 signaling.

Pyroptosis is a recognized form of regulated cell death that depends on caspase‐1 and inflammasome.^14^, ^15^ During the process of pyroptosis, ASC binds with NLRP3 and pro‐caspase1 to mediate the activation of caspase‐1.^16^ Recent evidence suggested that pyroptosis of macrophages can be stimulated by LPS treatment,^17^ thus, we used LPS to establish the cell model. In this study, consistent with these past studies, we also found that LPS induces pyroptosis of RAW264.7 macrophages.

Citrulline, as a precursor of arginine synthesis, is a potential immunomodulator.^18^ Citrulline was reported to possess anti‐inflammatory effects. It is reported to possess anti‐inflammatory effects by suppressing pyroptosis. A previous study has revealed that citrulline inhibits pyroptosis, apoptosis, and inflammatory changes induced by LPS in lung.^19^ Consistently, the results in this study indicated that citrulline promoted LPS‐induced cell viability and suppressed pyroptosis of RAW264.7 cells.

In recent years, many signaling pathways are involved in the regulation of pyroptosis. For example, the HMGB1/RAGE signaling pathway is involved in the regulation of pyroptosis of coronary artery endothelial cells and cervical epithelial cells.^20^, ^21^ The NLRP3/caspase‐1 signaling pathway regulates pyroptosis in a variety of diseases including sepsis, triple‐negative breast cancer, and myocardial ischemia/reperfusion.^22^, ^23^, ^24^ Meanwhile, the NF‐κB signaling pathway has also been reported to be involved in cell pyroptosis.^25^, ^26^, ^27^ LPS is known to activate the NF‐κB pathway, which regulates inflammatory responses by regulating a variety of pro‐inflammatory cytokines in macrophages.^28^, ^29^, ^30^ In the current work, LPS was confirmed to activate the NF‐κB pathway in RAW264.7 macrophages to promote pyroptosis.

Exogenous citrulline regulates NO synthesis through a negative feedback effect, which can alleviate the damage of important organs such as the heart, liver, and kidney.^31^, ^32^ The NF‐κB pathway may be the underlying mechanism of citrulline function. For example, Man et al. have revealed that citrulline attenuates the progression of pre‐eclampsia by suppressing the NF‐κB pathway.^33^ In addition, Ba et al. have identified that citrulline inhibits ferroptosis in the thymus via inactivating the NF‐κB pathway.^34^ Herein, we predicted that citrulline could combine with p65. However, whether citrulline mediated the NF‐κB pathway to participate in pyroptosis in macrophages. Our finding illustrated that citrulline inhibited the nuclear translocation of p65 and inhibited the NF‐κB pathway activation.

The major limitation of this study is that we did not explore whether citrulline affected macrophage pyroptosis in vivo. We will study the effects of citrulline on inflammation by regulating macrophage pyroptosis in animal models in our future work.

## 5 CONCLUSION

In conclusion, LPS‐promoted pyroptosis of macrophages. Citrulline inhibits LPS‐induced macrophage pyroptosis by inactivating the NF‐κB pathway mediated by p65 nuclear translocation. These findings might provide a novel selection for inflammatory diseases.

## AUTHOR CONTRIBUTIONS

Li Yin: Conceptualization (lead); writing—original draft (lead); formal analysis (lead); writing—review and editing (equal). Xiaomin Wei: Software (lead); writing—review and editing (equal). Yanjun Zhang and Chengshu Lu: Methodology (lead); writing—review and editing (equal). Huakun Wang: Conceptualization (supporting); writing—original draft (supporting); writing—review and editing (equal).

## CONFLICT OF INTEREST STATEMENT

The authors declare no conflict of interest.

## Data Availability

The datasets used and analyzed during the current study are available from the corresponding author on reasonable request.
